# The Value of Serial Measurement of Arterial Stiffness in Cardiovascular Disease

**DOI:** 10.31083/j.rcm2501007

**Published:** 2024-01-08

**Authors:** Hack-Lyoung Kim

**Affiliations:** ^1^Division of Cardiology, Department of Internal Medicine, Seoul Metropolitan Government-Seoul National University Boramae Medical Center, Seoul National University College of Medicine, 07061 Seoul, Republic of Korea

**Keywords:** arterial stiffness, pulse wave velocity, repeated measurements, serial measurements

## Abstract

Clinically assessing arterial stiffness is valuable because it aids in predicting future cardiovascular events. There are several methods for measuring arterial stiffness, including pulse wave velocity (PWV), augmentation index, and pulse pressure. Numerous studies have shown that these indicators of arterial stiffness possess prognostic value for various patient groups as well as the general population. In cross-sectional studies, arterial stiffness was also linked to organ damage indices. However, most studies related to arterial stiffness have relied on a single measurement. Taking multiple serial measurements of arterial stiffness offers several advantages. Through repeated assessments, one can confirm the variability of arterial stiffness and observe changes over time, which is beneficial for understanding its pathophysiology. Such repeated measurements are also invaluable in evaluating the efficacy of interventions aimed at improving arterial stiffness. However, caution is needed, as there is no standardized method for measuring arterial stiffness. For instance, with PWV, the values can be influenced by numerous external factors. Therefore, the external conditions during the measurement must be noted. It’s essential to recognize the pros and cons of repeated arterial stiffness measurements and integrate them effectively into clinical practice.

## 1. Introduction

The stiffness of artery walls naturally increases as part of the aging process [1, 2]. However, this arterial stiffness is not solely the result of aging. Several other factors also contribute to its augmentation, such as a prolonged exposure to high blood pressure (BP), hyperglycemia, smoking, inflammation and reactive oxygen species [3, 4]. All these factors together lead to a more rigid arterial system, consequently impacting the overall cardiovascular health of an individual. This rise in arterial stiffness is a crucial factor closely associated with future cardiovascular events. It has been observed that individuals with stiffer arteries are at a heightened risk of cardiovascular incidents [5, 6]. In addition, arterial stiffness is also a strong predictor of potential target organ damage [7, 8, 9]. Thus, monitoring arterial stiffness is a useful tool for predicting future cardiovascular events, not only in specific patient groups who are already at risk [10, 11, 12, 13, 14, 15, 16] but also in the general population [17, 18, 19].

By periodically and repeatedly measuring the level of arterial stiffness at regular intervals, clinicians can obtain a more detailed and nuanced understanding of an individual’s cardiovascular health. This can inform a more comprehensive, proactive approach to disease prevention and management [11, 20, 21, 22]. However, most of the current research on arterial stiffness is based on a single measurement taken at a specific point in time, which may not provide a complete picture of the individual’s ongoing cardiovascular condition. Repeated measurements of arterial stiffness offer valuable data, but their use in clinical practice presents a unique set of challenges. Some of these issues include the logistical difficulties associated with frequent testing, patient compliance, and the variability in measurements due to multiple factors. These challenges may have hindered the progress of research in this area.

This review aims to explore the advantages and disadvantages of using repeated measurements of arterial stiffness as a tool in the field of cardiovascular disease (CVD). It will shed light on how these measurements can be effectively used, the obstacles to their implementation, and possible solutions to overcome these challenges. This deeper understanding of arterial stiffness and its implications will be vital in driving improvements in cardiovascular care.

## 2. How to Measure Arterial Stiffness

There are several methods to measure arterial stiffness. The most commonly used method, both clinically and in research, is the measurement of pulse wave velocity (PWV) [23]. PWV measures the speed of the pulse wave traveling along the arterial wall. It operates on the principle that as arterial stiffness increases, the speed of the pulse wave increases correspondingly [24]. Generally, PWV is calculated by dividing the distance between two points by the time difference between the pulse wave arrivals at these points [23]. Several types of PWV exist, depending on the two points chosen for measurement. The most widely recognized types are carotid-femoral PWV (cfPWV) and brachial-ankle PWV (baPWV) [25]. Since cfPWV includes only elastic arteries, it is theoretically the most accurate indicator of central artery stiffness [26]. cfPWV has historically been the first method used and its prognostic value has been validated in numerous clinical studies. It is considered the gold standard for non-invasive measurements of arterial stiffness [26]. baPWV, a method developed in Japan following cfPWV, is primarily used in Asian populations [27]. Since baPWV incorporates muscular arteries, it is not as precise a measure of central artery stiffness as cfPWV [27]. However, it provides a more comprehensive representation of the heart’s total afterload [28]. Unlike cfPWV, which requires technical skill to locate the carotid and femoral arteries, baPWV measurement is more straightforward and convenient, as it only involves placing a BP cuff around the arm or leg. This convenience makes baPWV advantageous when measuring a large number of patients [4].

PWV is widely utilized due to its non-invasive nature and the relative ease with which it can be measured. However, one of the biggest disadvantages of PWV is that the PWV value is greatly affected by BP. As BP rises, the PWV value also increases, so the BP value must be corrected or considered when interpreting the PWV value [29]. To overcome these shortcomings of PWV, alternative indices like β-stiffness index and cardio-ankle vascular index (CAVI), not affected by BP at the time of measurement, were proposed. The β-stiffness index, an index reflecting arterial stiffness of local arterial segment, quantifies the change in arterial diameter in response to a change in BP and is represented as = ln (systolic BP/diastolic BP)/(arterial diameter at systole – arterial diameter at diastole) × arterial diameter at diastole. This index acts as a measure of arterial elasticity or compliance [30]. A higher value of the β-stiffness index indicates greater arterial stiffness, suggesting the arteries are less able to expand and contract with each heartbeat, potentially impairing blood flow and increasing cardiovascular risk. CAVI, developed in 2004, represents arterial stiffness from the origin of the aorta to the ankle and is also computed based on the change in arterial wall diameter due to BP fluctuations from Bramwell-Hill’s equation—this being the fundamental principle behind the calculation of the β-stiffness index [31].

Another method to assess arterial stiffness involves measuring parameters related to wave reflection. As arterial stiffness increases, the velocity of these reflected waves also increases, causing them to merge with the forward-traveling wave sooner. This leads to an increase in systolic pressure (= augmentation pressure (AP)) and a decrease in diastolic pressure, which in turn results in a heightened pulse pressure (PP) [32, 33]. Thus, an elevation in AP and PP can serve as indicators of arterial stiffness [33]. Augmentation index (AIx) is calculated as the difference between the reflected and forward systolic peaks (= AP), expressed as a percentage of the PP (= AP/PP × 100%). A higher AIx value indicates the premature return of the reflected wave, which is typically attributed to increased arterial stiffness [34].

Arterial compliance and arterial stiffness can be assessed by observing changes in arterial diameter during systole and diastole. These measurements can be made using imaging technologies such as ultrasound, computed tomography, and magnetic resonance imaging (MRI). MRI provides high-resolution images, allowing detailed analysis of the arterial wall and its components. The ability to visualize and quantify multiple parameters related to arterial structure and function enables a more comprehensive assessment of arterial stiffness. MRI can measure aortic distensibility (= change in aortic area/[aortic area at diastole × PP]), which is a direct marker of arterial stiffness [35]. However, imaging studies measuring arterial stiffness are not widely used due to several limitations. Firstly, the accuracy of these measurements can be relatively low. Secondly, they require specialized, expensive equipment, as well as substantial time for both conducting the test and interpreting the results. Additionally, obtaining high-quality images is essential for reliable measurements, which adds another layer of complexity to this method [23].

PWV, AP and PP can be assessed through invasive measurement of aortic pressure. This involves the insertion of a catheter directly into the aortic root to measure intra-arterial pressure. Although this method provides highly accurate results, it is invasive and is generally limited to patients who are already undergoing invasive procedures like coronary angiography [10, 36]. With technological advancements, it’s now possible to estimate central arterial pressure by analyzing the waveform of the radial artery. This method is known for its excellent accuracy and is becoming increasingly utilized in clinical practice [37, 38].

## 3. Arterial Stiffness and Cardiovascular Risk

Previous studies have established a close association between increased arterial stiffness and organ damage. Specifically, it has been confirmed that patients with increased arterial stiffness exhibit a higher incidence of left ventricular hypertrophy [8], left ventricular diastolic dysfunction [7, 39], coronary artery stenosis [40], and cerebrovascular disease [41]. Furthermore, arterial stiffness has been found to be a powerful and reliable predictor of future cardiovascular events. The implications of this discovery are far-reaching, applying not only to the general population [17, 18, 19] but even extending to patients with certain diseases [10, 11, 12, 13, 14, 15, 16]. Further reinforcing these findings, a comprehensive meta-analysis of numerous studies has unequivocally reasserted the prognostic value of arterial stiffness in anticipating cardiovascular events [5, 6]. Intriguingly, it has been found that the predictive potential of arterial stiffness is independent of traditional cardiovascular risk factors. This includes well-established risk contributors such as hypertension, diabetes, obesity, and smoking habits. Such findings suggest the importance of arterial stiffness in stratifying cardiovascular risk across diverse patient cohorts.

## 4. Pathophysiology

Several reasons have been proposed to explain the heightened risk of organ damage and poor cardiovascular prognosis associated with increased arterial stiffness. Blood vessels that exhibit this heightened stiffness can’t properly buffer the pulsatile energy from the heart, which can lead to target organ damage [33]. Moreover, an increase in arterial stiffness accelerates the speed of the reflected wave, causing it to encounter the forward wave at an earlier stage. This interaction leads to a rise in systolic BP and a drop in diastolic BP. The ensuing increase in both systolic BP and PP can trigger left ventricular hypertrophy, which in turn promotes left ventricular diastolic dysfunction and subendocardial ischemia [4]. A decrease in diastolic BP can reduce coronary blood flow [32]. Additionally, an increased PP can disrupt the blood-brain barrier, potentially causing brain damage. Factors such as aging, high BP, inflammation, and oxidative stress often accompany increased arterial stiffness and can accelerate the progression of atherosclerosis [4].

## 5. Serial Measurement of Arterial Stiffness

Most studies on arterial stiffness have relied on a single measurement of the arterial stiffness index at a specific time point. However, data derived from repeated measurements of arterial stiffness can yield more informative insights. The process of taking serial measurements of arterial stiffness carries several significant advantages that can greatly benefit medical research and patient management. For instance, fluctuations in arterial stiffness over time can serve as early indicators of vascular diseases like atherosclerosis [42, 43], thus enabling timely interventions. Additionally, repeated measurements of arterial stiffness enable the identification of pathophysiology more clearly related to arterial stiffness, which may not be adequately understood through a single measurement [20, 44, 45, 46, 47, 48, 49]. Furthermore, through the serial measurement of arterial stiffness, physicians can evaluate the efficacy of therapeutic interventions on a patient’s arterial health [50, 51, 52, 53, 54, 55, 56, 57, 58, 59, 60, 61]. A treatment that leads to a reduction in arterial stiffness often signals its effectiveness. Additionally, serial measurements of arterial stiffness can aid in stratifying the risk of future cardiovascular events in patients. This tool is invaluable for physicians as it allows them to identify individuals at high risk and make more informed decisions regarding their treatment plans. Finally, serial measurements can prove useful in tracking disease progression [20]. An increase in arterial stiffness may signify deteriorating health, while a decrease or stability in stiffness may indicate health improvement or maintenance of stability [43, 62]. Table 1 (Ref. [20, 42, 43, 44, 45, 46, 47, 48, 49, 50, 51, 52, 53, 54, 55, 56, 57, 58, 59, 60, 61, 62, 63, 64, 65, 66]) summarizes the results of several major studies related to serial measurement of arterial stiffness.

**Table 1.** **Summary of several clinical studies on the serial measurements of arterial stiffness**.

Source (year)	Study population	Number of subjects	Intervention	Time interval between the measurements	Measure of arterial stiffness	Main result
Kim *et al*. (2023) [62]	Patients underwent PCI	405	No interventions	1 month	baPWV	Increased baPWV/SBP was associated with worse clinical outcomes.
Zhou *et al*. (2022) [54]	Adults with higher atherosclerotic risk	820	Statins	Mean 4.8 years	baPWV	Compared with non-statin users, statin users had significantly slower progression of baPWV.
Nakamura *et al*. (2021) [53]	Patients with CAD	323	Optimal medical treatment	2 years	baPWV	Improvement of baPWV was associated with better cardiovascular outcome.
Rueangjaroen *et al*. (2021) [46]	Pregnant women	335	No intervention	Gestational age 11–14, 18–22, 28–32, and after 36 weeks	CAVI	Increased CAVI was associated with the development of preeclampsia and fetal growth restriction.
Mandraffino *et al*. (2020) [59]	Patients with FH	98	PCSK-9 inhibitor or ezetimibe	6 months	cfPWV	Add on therapy of PCSK-9 inhibitor or ezetimibe to statin therapy significantly reduced cfPWV.
Toussaint *et al*. (2020) [55]	Patients with stage 3b or 4 CKD	278	Phosphate-lowering medication	96 weeks	cfPWV	cfPWV was not changed by phosphate-lowering medication.
Kim *et al*. (2020) [63]	Patients with preeclampsia	37	No intervention	1 year	CAVI	CAVI was not changed at 1 year after preeclampsia.
Reshetnik *et al*. (2020) [64]	ESRD patients	54	Dialysis	7 days	aPWV	aPWV was not changed by dialysis.
Jennings *et al*. (2019) [50]	Healthy subjects	225	Mediterranean-style diet	1 year	cfPWV, AIx	cfPWV was not changed but AIx decreased by Mediterranean-style diet.
Kadoya *et al*. (2018) [45]	General population	306	No intervention	3 years	baPWV	Low sleep quality was associated with increased baPWV.
Merlocco *et al*. (2017) [47]	Children and young adults with connective tissue disorders	50	No intervention	Median 3.9 years	CMR	There was a weak correlation between increased arterial stiffness and aortic root dilatation.
Kong *et al*. (2017) [49]	Healthy subjects	7154	No intervention	3 years	cfPWV	Increased cfPWV was associated with CKD development.
Peyster *et al*. (2017) [44]	CKD patients	2933	No intervention	0, 2, 4 years	cfPWV	cfPWV change was not associated with baseline levels of inflammatory markers.
Yuan *et al*. (2016) [56]	Young male overweight adults	20	Swimming	8 weeks	β-stiffness index, carotid artery	Carotid arterial stiffness decreased after exercise.
Ro *et al*. (2016) [51]	K-TPL recipients	67	K-TPL	6 month, 1, 2 years	baPWV	baPWV was improved by K-TPL.
Seetho *et al*. (2015) [57]	Patients with obesity and OSA	52	CPAP	Median 13.5 months	AIx	AIx was reduced by CPAP.
Jochemsen *et al*. (2015) [48]	Patients with arterial disease	526	No intervention	Mean 4.1 years	CMR	Carotid artery stiffening was not associated with brain volumes or infarcts.
Otsuka *et al*. (2014) [43]	Patients with CAD	211	No intervention	6 months	CAVI	Persistent impairment of arterial stiffness was an independent risk factor of future CVD events.
Oberoi *et al*. (2013) [42]	Patients with suspected CAD	164	No intervention	Mean 12 months	Aortic distensibility index by CT	The progression of aortic stiffness is associated with the progression of coronary atherosclerosis.
AlGhatrif *et al*. (2013) [20]	General population	777	No intervention	2~9 serial measurement between 1988 and 2013	cfPWV	There was a steeper longitudinal increase of cfPWV in men than women.
Kim *et al*. (2011) [60]	Patients with diabetes and hypertension	47	Valsartan	12 weeks	cfPWV	Valsartan improved cfPWV.
Phillips *et al*. (2010) [52]	Healthy male subjects	28	High-fat meal diet	6 hours	AIx	AIx decreased by high-fat meal diet.
Eryılmaz *et al*. (2010) [61]	Patients with OSA	44	CPAP	6 months	Arterial elasticity indices	CPAP therapy improves arterial elasticity.
Yoon *et al*. (2010) [65]	Healthy subjects	13	Resistance exercise	At baseline and 20 minutes after exercise	cfPWV, AIx	cfPWV and AIx were increased in after resistance exercise.
Yokoyama *et al*. (2005) [58]	Patients with hyperlipidemia	40	Fluvastatin	12 months	baPWV	Fluvastin decreased baPWV value.
Rajzer *et al*. (2003) [66]	Hypertensive subjects	118	Amlodipine, losartan, quinapril	0, 3, 6 months of medications	cfPWV	Only quinapril was associated with cfPWV reduction.

PCI, percutaneous coronary intervention; CAD, coronary artery disease; FH, familial hypercholesterolemia; CKD, chronic kidney disease; ESRD, end-stage renal disease; K-TPL, kidney transplantation; OSA, obstructive sleep apnea; CPAP, Continuous Positive Airway Pressure; baPWV, brachial-ankle PWV; CAVI, cardio-ankle vascular index; cfPWV, carotid-femoral PWV; aPWV, arterial pulse wave velocity; AIx, Augmentation index; CMR, cardiac magnetic resonance; CT, computerized tomography; SBP, Spontaneous Bacteria Peritonitis; CVD, cardiovascular disease.

## 6. Clinical Implication of the Serial Measurement of Arterial Stiffness

By undertaking serial measurements, clinicians can monitor the trajectory of arterial stiffness in individual patients [20, 67, 68, 69]. Such longitudinal assessments allow for detecting subtle changes that may precede overt symptoms or clinical manifestations of CVD [20, 68]. This proactive monitoring allows for timely interventions, such as early identification of a gradual rise in BP or incremental changes in blood glucose levels [69, 70]. Early detection opens the way for personalized therapeutic strategies encompassing lifestyle modifications, dietary changes, and pharmacological interventions, potentially halting or even reversing arterial deterioration [54, 56]. Moreover, serial measurements aid in evaluating the efficacy of treatments [58, 60, 61, 66]. By monitoring the parameters of arterial stiffness, clinicians can determine the therapeutic efficacy of a chosen strategy and make necessary adjustments. Additionally, these repetitive assessments enable personalized patient care. As every individual responds differently to treatments, frequent measurements allow tailoring therapy based on real-time data, leading to optimal outcomes. Finally, understanding the progression of CVD through serial measurements provides valuable prognostic information. Patients showing consistent improvements in measured parameters have a better prognosis than those with deteriorating or fluctuating values [43, 62].

## 7. Limitations of the Serial Measurement of Arterial Stiffness

Although serial measurement of arterial stiffness has become an invaluable tool in both cardiovascular research and clinical practice, it has several shortcomings. Various devices and techniques, such as tonometry, Doppler, and oscillometry, can produce slightly different arterial stiffness values [23]. Furthermore, the measurements can be operator-dependent, introducing potential variability. Arterial stiffness is subject to fluctuations based on factors like circadian rhythms, dietary intake, stress, and other transient influences, affecting the timing and consistency of measurements [26]. Especially with PWV, the values are sensitive to current BP [29]. Distinguishing between inherent changes in the arterial wall and just BP alterations can be challenging. Additionally, arterial stiffness is not uniform throughout the body. For instance, while cfPWV evaluates aortic stiffness, it may not accurately represent the stiffness in peripheral or coronary arteries. Standardized values for arterial stiffness across diverse populations are lacking, complicating the establishment of definitive risk thresholds. Moreover, the cost and accessibility of regular measurements can be prohibitive, limiting their widespread use in some clinical settings. Factors like arrhythmias or pronounced obesity can hinder the acquisition of precise PWV measurements. Therefore, while serial measurements of arterial stiffness provide significant insights, their interpretation requires careful consideration of the aforementioned constraints. To maximize their efficacy in cardiovascular risk management, these measurements should be incorporated alongside other clinical and investigatory data.

The strengths and limitations of serial measurement of arterial stiffness are summarized in Table 2.

**Table 2.** **The strengths and limitations of serial measurements of arterial stiffness**.

Strengths	Limitations
Monitoring the trajectory	Device and technique variability
Allows for timely interventions	Operator-dependence
Personalized therapeutic strategies	Sensitivity to many clinical factors at the time of measurement
Evaluating efficacy of treatments	Lack of standardized values across populations
Provides valuable prognostic information	Cost and accessibility

## 8. Conclusions

Serial measurements of arterial stiffness significantly impact the management and prognosis of CVD by offering a comprehensive, evolving view of cardiovascular health, thereby guiding effective interventions and improving patient outcomes. However, it is essential to note that while these serial measurements are insightful, they must be interpreted with their limitations in mind. To maximize their effectiveness in cardiovascular risk management, it is beneficial to incorporate these measurements alongside other clinical and research data.

## References

[1] Mitchell GF (2021). Arterial Stiffness in Aging: Does It Have a Place in Clinical Practice ?: Recent Advances in Hypertension. *Hypertension*.

[2] Lu Y, Kiechl SJ, Wang J, Xu Q, Kiechl S, Pechlaner R (2023). Global distributions of age- and sex-related arterial stiffness: systematic review and meta-analysis of 167 studies with 509,743 participants. *EBioMedicine*.

[3] Chirinos JA, Segers P, Hughes T, Townsend R (2019). Large-Artery Stiffness in Health and Disease. *Journal of the American College of Cardiology*.

[4] Kim HL, Kim SH (2019). Pulse Wave Velocity in Atherosclerosis. *Frontiers in Cardiovascular Medicine*.

[5] Vlachopoulos C, Aznaouridis K, Stefanadis C (2010). Prediction of Cardiovascular Events and all-Cause Mortality with Arterial Stiffness. *Journal of the American College of Cardiology*.

[6] Ohkuma T, Ninomiya T, Tomiyama H, Kario K, Hoshide S, Kita Y (2017). Brachial-Ankle Pulse Wave Velocity and the Risk Prediction of Cardiovascular Disease. *Hypertension*.

[7] Kim M, Kim HL, Lim WH, Seo JB, Kim SH, Kim MA (2022). Association between arterial stiffness and left ventricular diastolic function: A large population-based cross-sectional study. *Frontiers in Cardiovascular Medicine*.

[8] Kwak S, Kim H, Lim W, Seo J, Kim S, Zo J (2022). Sex-specific associations of brachial–ankle pulse wave velocity with adverse cardiac remodeling and long-term cardiovascular outcome. *Journal of Hypertension*.

[9] Vasan RS, Short MI, Niiranen TJ, Xanthakis V, DeCarli C, Cheng S (2019). Interrelations between Arterial Stiffness, Target Organ Damage, and Cardiovascular Disease Outcomes. *Journal of the American Heart Association*.

[10] Hametner B, Wassertheurer S, Mayer CC, Danninger K, Binder RK, Weber T (2021). Aortic Pulse Wave Velocity Predicts Cardiovascular Events and Mortality in Patients Undergoing Coronary Angiography. *Hypertension*.

[11] Moh MC, Sum CF, Tavintharan S, Ang K, Lee SBM, Tang WE (2017). Baseline predictors of aortic stiffness progression among multi-ethnic Asians with type 2 diabetes. *Atherosclerosis*.

[12] Demir S, Akpınar O, Akkus O, Nas K, Unal I, Molnar F (2013). The prognostic value of arterial stiffness in systolic heart failure. *Cardiology Journal*.

[13] Lee Y, Park J, Kim E, Kang C, Park H (2014). Arterial Stiffness and Functional Outcome in Acute Ischemic Stroke. *Journal of Cerebrovascular and Endovascular Neurosurgery*.

[14] Ishizuka K, Hoshino T, Shimizu S, Shirai Y, Mizuno S, Toi S (2016). Brachial-ankle pulse wave velocity is associated with 3-month functional prognosis after ischemic stroke. *Atherosclerosis*.

[15] Szeto C, Kwan BC, Chow K, Leung C, Law M, Li PK (2012). Prognostic Value of Arterial Pulse Wave Velocity in Peritoneal Dialysis Patients. *American Journal of Nephrology*.

[16] Kirigaya J, Iwahashi N, Tahakashi H, Minamimoto Y, Gohbara M, Abe T (2020). Impact of Cardio-Ankle Vascular Index on Long-Term Outcome in Patients with Acute Coronary Syndrome. *Journal of Atherosclerosis and Thrombosis*.

[17] Willum Hansen T, Staessen JA, Torp-Pedersen C, Rasmussen S, Thijs L, Ibsen H (2006). Prognostic Value of Aortic Pulse Wave Velocity as Index of Arterial Stiffness in the General Population. *Circulation*.

[18] Mattace-Raso FUS, van der Cammen TJM, Hofman A, van Popele NM, Bos ML, Schalekamp MADH (2006). Arterial Stiffness and Risk of Coronary Heart Disease and Stroke. *Circulation*.

[19] Miyoshi T, Ito H, Shirai K, Horinaka S, Higaki J, Yamamura S (2021). Predictive Value of the Cardio-Ankle Vascular Index for Cardiovascular Events in Patients at Cardiovascular Risk. *Journal of the American Heart Association*.

[20] AlGhatrif M, Strait JB, Morrell CH, Canepa M, Wright J, Elango P (2013). Longitudinal Trajectories of Arterial Stiffness and the Role of Blood Pressure. *Hypertension*.

[21] Roderjan CN, Cardoso CRL, Ferreira MT, Muxfeldt ES, Salles GF (2015). Correlates of aortic stiffness progression in patients with resistant hypertension. *Journal of Hypertension*.

[22] Ferreira MT, Leite NC, Cardoso CRL, Salles GF (2015). Correlates of Aortic Stiffness Progression in Patients with Type 2 Diabetes: Importance of Glycemic Control. *Diabetes Care*.

[23] Cavalcante JL, Lima JAC, Redheuil A, Al-Mallah MH (2011). Aortic Stiffness. *Journal of the American College of Cardiology*.

[24] Salvi P, Magnani E, Valbusa F, Agnoletti D, Alecu C, Joly L (2008). Comparative study of methodologies for pulse wave velocity estimation. *Journal of Human Hypertension*.

[25] Park JB, Sharman JE, Li Y, Munakata M, Shirai K, Chen C (2022). Expert Consensus on the Clinical Use of Pulse Wave Velocity in Asia. *Pulse*.

[26] Laurent S, Cockcroft J, Van Bortel L, Boutouyrie P, Giannattasio C, Hayoz D (2006). Expert consensus document on arterial stiffness: methodological issues and clinical applications. *European Heart Journal*.

[27] Sugawara J, Tanaka H (2015). Brachial-Ankle Pulse Wave Velocity: Myths, Misconceptions, and Realities. *Pulse*.

[28] Chow B, Rabkin SW (2013). Brachial-Ankle Pulse Wave Velocity is the only Index of Arterial Stiffness that Correlates with a Mitral Valve Indices of Diastolic Dysfunction, but no Index Correlates with Left Atrial Size. *Cardiology Research and Practice*.

[29] (2010). Determinants of pulse wave velocity in healthy people and in the presence of cardiovascular risk factors: ‘establishing normal and reference values’. *European Heart Journal*.

[30] Hayashi K, Yamamoto T, Takahara A, Shirai K (2015). Clinical assessment of arterial stiffness with cardio-ankle vascular index. *Journal of Hypertension*.

[31] Shirai K, Utino J, Otsuka K, Takata M (2006). A Novel Blood Pressure-independent Arterial Wall Stiffness Parameter; Cardio-Ankle Vascular Index (CAVI). *Journal of Atherosclerosis and Thrombosis*.

[32] Kim HL, Weber T (2021). Pulsatile Hemodynamics and Coronary Artery Disease. *Korean Circulation Journal*.

[33] Dart AM, Kingwell BA (2001). Pulse pressure—a review of mechanisms and clinical relevance. *Journal of the American College of Cardiology*.

[34] Wilkinson IB, MacCallum H, Flint L, Cockcroft JR, Newby DE, Webb DJ (2000). The influence of heart rate on augmentation index and central arterial pressure in humans. *The Journal of Physiology*.

[35] Hrabak-Paar M, Kircher A, Al Sayari S, Kopp S, Santini F, Schmieder RE (2020). Variability of MRI Aortic Stiffness Measurements in a Multicenter Clinical Trial Setting: Intraobserver, Interobserver, and Intracenter Variability of Pulse Wave Velocity and Aortic Strain Measurement. *Radiology: Cardiothoracic Imaging*.

[36] Chung J, Kim H, Lim W, Seo J, Kim S, Zo J (2019). Association between invasively measured aortic pulse pressure and orthostatic hypotension in patients undergoing invasive coronary angiography. *Journal of Hypertension*.

[37] Sharman JE, Lim R, Qasem AM, Coombes JS, Burgess MI, Franco J (2006). Validation of a Generalized Transfer Function to Noninvasively Derive Central Blood Pressure during Exercise. *Hypertension*.

[38] Chen C, Nevo E, Fetics B, Pak PH, Yin FCP, Maughan WL (1997). Estimation of Central Aortic Pressure Waveform by Mathematical Transformation of Radial Tonometry Pressure. *Circulation*.

[39] Kim H-, Seo J-, Chung W-, Kim S-, Kim M-, Zo J- (2015). Association between Invasively Measured Central Aortic Pressure and Left Ventricular Diastolic Function in Patients Undergoing Coronary Angiography. *American Journal of Hypertension*.

[40] Kim HL, Jin KN, Seo JB, Choi YH, Chung WY, Kim SH (2015). The association of brachial-ankle pulse wave velocity with coronary artery disease evaluated by coronary computed tomography angiography. *PLoS ONE*.

[41] van Sloten TT, Protogerou AD, Henry RMA, Schram MT, Launer LJ, Stehouwer CDA (2015). Association between arterial stiffness, cerebral small vessel disease and cognitive impairment: a systematic review and meta-analysis. *Neuroscience and Biobehavioral Reviews*.

[42] Oberoi S, Schoepf UJ, Meyer M, Henzler T, Rowe GW, Costello P (2013). Progression of Arterial Stiffness and Coronary Atherosclerosis: Longitudinal Evaluation by Cardiac CT. *American Journal of Roentgenology*.

[43] Otsuka K, Fukuda S, Shimada K, Suzuki K, Nakanishi K, Yoshiyama M (2014). Serial assessment of arterial stiffness by cardio-ankle vascular index for prediction of future cardiovascular events in patients with coronary artery disease. *Hypertension Research*.

[44] Peyster E, Chen J, Feldman HI, Go AS, Gupta J, Mitra N (2017). Inflammation and Arterial Stiffness in Chronic Kidney Disease: Findings from the CRIC Study. *American Journal of Hypertension*.

[45] Kadoya M, Kurajoh M, Kakutani-Hatayama M, Morimoto A, Miyoshi A, Kosaka-Hamamoto K (2018). Low sleep quality is associated with progression of arterial stiffness in patients with cardiovascular risk factors: HSCAA study. *Atherosclerosis*.

[46] Rueangjaroen P, Luewan S, Phrommintikul A, Leemasawat K, Tongsong T (2021). The cardio-ankle vascular index as a predictor of adverse pregnancy outcomes. *Journal of Hypertension*.

[47] Merlocco A, Lacro RV, Gauvreau K, Rabideau N, Singh MN, Prakash A (2017). Longitudinal Changes in Segmental Aortic Stiffness Determined by Cardiac Magnetic Resonance in Children and Young Adults with Connective Tissue Disorders (the Marfan, Loeys-Dietz, and Ehlers-Danlos Syndromes, and Nonspecific Connective Tissue Disorders). *The American Journal of Cardiology*.

[48] Jochemsen HM, Muller M, Bots ML, Scheltens P, Vincken KL, Mali WPTM (2015). Arterial stiffness and progression of structural brain changes: the SMART-MR study. *Neurology*.

[49] Kong X, Ma X, Tang L, Wang Z, Li W, Cui M (2017). Arterial stiffness evaluated by carotid‐femoral pulse wave velocity increases the risk of chronic kidney disease in a Chinese population‐based cohort. *Nephrology*.

[50] Jennings A, Berendsen AM, de Groot LCPGM, Feskens EJM, Brzozowska A, Sicinska E (2019). Mediterranean-Style Diet Improves Systolic Blood Pressure and Arterial Stiffness in Older Adults. *Hypertension*.

[51] Ro H, Kim AJ, Chang JH, Jung JY, Chung WK, Park YH (2016). Can Kidney Transplantation Improve Arterial Stiffness in End-Stage Renal Patients. *Transplantation Proceedings*.

[52] Phillips LK, Peake JM, Zhang X, Hickman IJ, Kolade O, Sacre JW (2010). The Effect of a High-Fat Meal on Postprandial Arterial Stiffness in Men with Obesity and Type 2 Diabetes. *The Journal of Clinical Endocrinology and Metabolism*.

[53] Nakamura T, Uematsu M, Horikoshi T, Yoshizaki T, Kobayashi T, Saito Y (2021). Improvement in Brachial Endothelial Vasomotor Function and Brachial-Ankle Pulse Wave Velocity Reduces the Residual Risk for Cardiovascular Events after Optimal Medical Treatment in Patients with Coronary Artery Disease. *Journal of Atherosclerosis and Thrombosis*.

[54] Zhou Y, Wang Y, Wang G, Zhou Z, Chen S, Geng T (2022). Association between Statin Use and Progression of Arterial Stiffness among Adults with High Atherosclerotic Risk. *JAMA Network Open*.

[55] Toussaint ND, Pedagogos E, Lioufas NM, Elder GJ, Pascoe EM, Badve SV (2020). A Randomized Trial on the Effect of Phosphate Reduction on Vascular End Points in CKD (IMPROVE-CKD). *Journal of the American Society of Nephrology*.

[56] Yuan W, Liu H, Gao F, Wang Y, Qin K (2016). Effects of 8-week swimming training on carotid arterial stiffness and hemodynamics in young overweight adults. *BioMedical Engineering OnLine*.

[57] Seetho IW, Asher R, Parker RJ, Craig S, Duffy N, Hardy KJ (2015). Effect of CPAP on arterial stiffness in severely obese patients with obstructive sleep apnoea. *Sleep and Breathing*.

[58] Yokoyama H, Kawasaki M, Ito Y, Minatoguchi S, Fujiwara H (2005). Effects of Fluvastatin on the Carotid Arterial Media as Assessed by Integrated Backscatter Ultrasound Compared with Pulse-Wave Velocity. *Journal of the American College of Cardiology*.

[59] Mandraffino G, Scicali R, Rodríguez-Carrio J, Savarino F, Mamone F, Scuruchi M (2020). Arterial stiffness improvement after adding on PCSK9 inhibitors or ezetimibe to high-intensity statins in patients with familial hypercholesterolemia: a Two–Lipid Center Real-World Experience. *Journal of Clinical Lipidology*.

[60] Kim JH, Oh SJ, Lee JM, Hong EG, Yu JM, Han KA (2011). The effect of an Angiotensin receptor blocker on arterial stiffness in type 2 diabetes mellitus patients with hypertension. *Diabetes and Metabolism Journal*.

[61] Eryılmaz S, Aydinlar A, Senturk T, Ursavas A, Aydın Kaderli A, Özdemir B (2010). Continuous positive airway pressure therapy improves arterial elasticity in patients with obstructive sleep apnea. *Respiratory Medicine*.

[62] Kim H, Joh HS, Lim W, Seo J, Kim S, Zo J (2023). One-month changes in blood pressure-adjusted pulse wave velocity for predicting long-term cardiovascular outcomes in patients undergoing percutaneous coronary intervention. *Journal of Hypertension*.

[63] Kim S, Lim HJ, Kim JR, Oh KJ, Hong JS, Suh JW (2020). Longitudinal change in arterial stiffness after delivery in women with preeclampsia and normotension: a prospective cohort study. *BMC Pregnancy and Childbirth*.

[64] Reshetnik A, Wrobel D, Wirtz G, Tölle M, Eckardt K, van der Giet M (2020). True Arterial Stiffness does not Change between Dialysis Sessions during 1 Week in Outpatients on Intermitted Hemodialysis. *Kidney and Blood Pressure Research*.

[65] Yoon ES, Jung SJ, Cheun SK, Oh YS, Kim SH, Jae SY (2010). Effects of Acute Resistance Exercise on Arterial Stiffness in Young Men. *Korean Circulation Journal*.

[66] Rajzer M (2003). Effect of amlodipine, quinapril, andlosartan on pulse wave velocity andplasma collagen markers in patients withmild-to-moderate arterial hypertension. *American Journal of Hypertension*.

[67] Scuteri A, Morrell CH, Orrù M, Strait JB, Tarasov KV, Ferreli LAP (2014). Longitudinal Perspective on the Conundrum of Central Arterial Stiffness, Blood Pressure, and Aging. *Hypertension*.

[68] Deng X, Song Y, Han X, Chen X, Yang W, Wu S (2023). Brachial-ankle pulse wave velocity trajectories in a middle-aged population. *Frontiers in Cardiovascular Medicine*.

[69] Zheng M, Zhang X, Chen S, Song Y, Zhao Q, Gao X (2020). Arterial Stiffness Preceding Diabetes. *Circulation Research*.

[70] Wu S, Tian X, Chen S, Zhang Y, Zhang X, Xu Q (2023). Arterial stiffness and blood pressure in treated hypertension: a longitudinal study. *Journal of Hypertension*.

